# Image Encryption Algorithm Based on Dynamic Rhombus Transformation and Digital Tube Model

**DOI:** 10.3390/e27080874

**Published:** 2025-08-18

**Authors:** Xiaoqiang Zhang, Yupeng Song, Ke Huang

**Affiliations:** School of Information and Control Engineering, China University of Mining and Technology, Xuzhou 221116, China; ts23060144p31@cumt.edu.cn (Y.S.); ts23060037a31@cumt.edu.cn (K.H.)

**Keywords:** image encryption, chaotic system, dynamic rhombus transformation, Manhattan distance, dynamic diffusion

## Abstract

With the rapid advancement of information technology, as critical information carriers, images are confronted with significant security risks. To ensure the image security, this paper proposes an image encryption algorithm based on a dynamic rhombus transformation and digital tube model. Firstly, a two-dimensional hyper-chaotic system is constructed by combining the Sine map, Cubic map and May map. The analysis results demonstrate that the constructed hybrid chaotic map exhibits superior chaotic characteristics in terms of bifurcation diagrams, Lyapunov exponents, sample entropy, etc. Secondly, a dynamic rhombus transformation is proposed to scramble pixel positions, and chaotic sequences are used to dynamically select transformation centers and traversal orders. Finally, a digital tube model is designed to diffuse pixel values, which utilizes chaotic sequences to dynamically control the bit reversal and circular shift operations, and the exclusive OR operation to diffuse pixel values. The performance analyses show that the information entropy of the cipher image is 7.9993, and the correlation coefficients in horizontal, vertical, and diagonal directions are 0.0008, 0.0001, and 0.0005, respectively. Moreover, the proposed algorithm has strong resistance against noise attacks, cropping attacks, and exhaustive attacks, effectively ensuring the security of images during storage and transmission.

## 1. Introduction

Nowadays, the information technology is developing rapidly. Massive and diversified data and information are widely disseminated globally, and all aspects of people’s life, such as work, study and communication, are closely connected with the internet. In the digital era, as important data carriers, images carry a wealth of information and have a wide range of applications, covering many key areas, such as healthcare, transportation, finance and daily life [1,2]. However, due to the open nature of the internet, digital images face significant security risks during storage and transmission. Some image information involves personal privacy or critical fields such as politics, military, medicine and commerce. If they are illegally accessed, tampered with or leaked during transmission, it will cause incalculable losses [3,4]. Image encryption technology can convert plaintext images into unrecognizable noise-like images through specific encryption means to protect the security of digital image information [5].

Digital images have characteristics of large data volume, high redundancy, strong inter-pixel correlation, etc. However, traditional encryption methods, including the Advanced Encryption Standard (AES) and the Data Encryption Standard (DES), are originally designed for text encryption. Therefore, they often exhibit low efficiency and inadequate encryption performance [6,7,8]. Researchers and industry experts have been dedicated to the development of image encryption technologies and have proposed various encryption methods based on other theories, including chaos theory [9], DNA coding technology [10], memristive neural network architectures [11] and quantum cryptography [12], etc.

Chaotic systems are widely used in the field of image encryption due to a series of dynamical properties, such as initial value sensitivity, nonlinearity and unpredictability [13]. Chaotic systems are generally classified into one-dimensional (1D) chaotic systems and high-dimensional chaotic systems. High-dimensional chaotic systems have attracted extensive research interest from scholars due to their larger key space and enhanced security performance, which can effectively resist sophisticated attacks. Zheng et al. constructed a two-dimensional (2D) chaotic system by integrating the nonlinear characteristics of the Sine map, Gaussian map, and infinite collapse iterative map [14]. This system exhibits superior ergodicity, high unpredictability, and complex chaotic attractors. Unfortunately, the bifurcation diagram did not fill the entire area. Wang et al. proposed a 2D crossed hyperchaotic sine-modulation-logistic map, which exhibits excellent key sensitivity and resilience against diverse attacks [15]. However, it is not a hyperchaotic system and lacks sufficiently complex dynamical behaviors. Zhang et al. proposed a composite coupled chaotic system in which each subsystem is influenced by the outputs of other subsystems, and the control parameters vary with the state of each dimension, thereby providing enhanced resistance against parameter identification attacks [16]. Regrettably, its mathematical model is too complex, increasing the computational cost. To achieve superior chaotic characteristics across the entire range of control parameters and to rapidly generate chaotic sequences, we have designed a novel 2D hyperchaotic system.

Most image encryption algorithms are based on a scrambling-diffusion structure. The scrambling phase involves rearranging the pixel positions, making it difficult for attackers to identify the original pixel positions and the relationships between adjacent pixels [17]. Image scanning techniques are widely used in scrambling algorithms due to their high efficiency and correlation destroying ability. Demirtas proposed a U-shaped scanning method, which significantly reduces the correlation between adjacent elements in images [18]. Sun et al. proposed a V-shaped transformation method that can fully scramble the image and eliminate the correlation between pixels [19]. Wang et al. proposed an L-shaped scanning method that fully breaks the correlation between pixel values [20]. However, most of the existing scanning methods have the problems of relatively fixed scanning starting points and scanning methods, which are vulnerable. Therefore, to effectively disrupt the pixel positions, we designed a dynamic rhombus transformation that uses chaotic sequences to dynamically select transformation centers and traversal orders.

The diffusion phase is the process of enhancing the algorithm security by changing the pixel values [21]. The exclusive OR (XOR) operation is widely used in the diffusion phase due to its good efficiency and reversibility. Wang et al. proposed a misalignment diffusion so that pixel values and chaotic sequences no longer correspond one-to-one for XOR operations, which greatly increases the algorithm security [22]. Wang et al. proposed a bit-level encryption algorithm that converts into 8 bits, applies circular shift operations to the bits, and then performs an XOR operation with a chaotic sequence. This algorithm exhibits high security and robustness [23]. Deng et al. proposed a nonlinear diffusion algorithm that disrupts pixel ordering using the Josephus ring algorithm, combined with S-box substitution and XOR operations to achieve dynamic pixel diffusion [24]. Inspired by the above literature, a dynamic diffusion method based on a digital tube model is proposed, utilizing chaotic sequences to dynamically control bit inversion and circular shift operations, combined with the XOR operation.

This paper proposes an image encryption algorithm based on a dynamic rhombus transformation and digital tube model. The main contributions are listed as follows:

(1) A novel 2D Sine-Cubic-May hyperchaotic map (2D-SCMH) is designed. By nonlinearly coupling the Sine map, Cubic map, and May map and incorporating the modulo operation to confine the chaotic range, the 2D-SCMH is able to maintain stable hyperchaotic characteristics throughout the entire parameter space while completely eliminating periodic windows.
(2) To scramble pixel positions, a dynamic rhombus transformation is proposed by utilizing a chaotic sequence to dynamically select the transformation center and traversal order. This transformation ensures that the scrambling process exhibits stronger randomness and traverses the entire image.
(3) To diffuse pixel values, a digital tube model is designed by utilizing chaotic sequences to control bit inversion and cyclic shift operation, integrated with XOR operation. This model enhances the randomness and unpredictability of the encryption process. In addition, it enables parallel processing of multiple pixels to significantly reduce the encryption time.
(4) An image encryption algorithm is proposed based on the 2D-SCMH chaotic system, dynamic rhombus transformation and digital tube model. This algorithm provides strong protection for image information and effectively guarantees the security of image transmission and storage.

The remainder of this paper is organized as follows. Section 2 introduces a hyperchaotic system named 2D-SCMH and analyzes its performance. Section 3 presents the designed scrambling and diffusion schemes. Section 4 describes the proposed encryption algorithm and the corresponding decryption algorithm. Section 5 demonstrates the experimental results of the proposed algorithm. Section 6 conducts a comprehensive evaluation and algorithm analysis. Finally, Section 7 concludes the research work of this paper.

## 2. Chaotic System

### 2.1. Classic Chaotic Maps

#### 2.1.1. Sine Map

The Sine map is a 1D chaotic map, and its structure is relatively simple. It is defined by Equation (1) [25]:

(1) $$x_{n+1}=\lambda \sin(\pi x_{n}),$$

where *λ* is the control parameter. When λ ∈ (0.8722, 1] and *x_n_* ∈ (0, 1), the Sine map is in a chaotic state.

#### 2.1.2. Cubic Map

The Cubic map is a classic 1D chaotic map. It is defined by Equation (2) [26]:

(2) $$x_{n+1}=\rho x_{n}(1-x_{n}^{2}),$$

where *ρ* is the control parameter. The Cubic map is in a chaotic state when *ρ* ∈ (2.3, 3].

#### 2.1.3. May Map

The May map is a classic map proposed by Robert May. It is defined by Equation (3) [27]:

(3) $$x_{n+1}=x_{n}e^{\gamma \left(1-x_{n}\right)},$$

where *γ* ∈ (0, 5) is the control parameter. The May map is in a chaotic state when *x_n_* ∈ (0, 10.9).

### 2.2. Definition of 2D-SCMH

To achieve better chaotic sequences, a novel 2D hyperchaotic system named 2D-SCMH is constructed by composite-coupling the Sine map, Cubic map, and May map. It is defined by

(4) $$\left\{\begin{array}{l} x_{n+1}=\mathrm{mod}(\sin\left(\frac{\mu \pi }{y_{n}e^{\mu \left(1-y_{n}\right)}}\right)+\cos\left(\frac{\mu \pi }{y_{n}\left(1-y_{n}^{2}\right)}\right)+\pi e^{{\left(x_{n}+y_{n}\right)}^{2}},1)\\ y_{n+1}=\mathrm{mod}(\cos\left(\frac{\mu \pi }{x_{n}e^{\mu \left(1-x_{n}\right)}}\right)+\sin\left(\frac{\mu \pi }{x_{n}\left(1-x_{n}^{2}\right)}\right)+\pi e^{{\left(x_{n}+y_{n}\right)}^{2}},1) \end{array}\right.,$$

where *x_n_*, *y_n_* are the state variables, *mod*(·) is the modulo operation, and *μ* ∈ (0, +∞) is the control parameter.

### 2.3. Chaotic Performance Analysis

To evaluate the performance of 2D-SCMH, we used a series of analytical metrics including bifurcation diagrams, Lyapunov exponents (LE), Shannon entropy, sample entropy, sensitivity analysis, 0-1 test and NIST test. In addition, to further validate the performance of 2D-SCMH, we conducted comparisons with some similar existing maps, including 2D-CLSS [28], 2D-CLII [29] and 2D-ILM [4] for comparative analysis.

#### 2.3.1. Bifurcation Diagram

**Figure 1:**
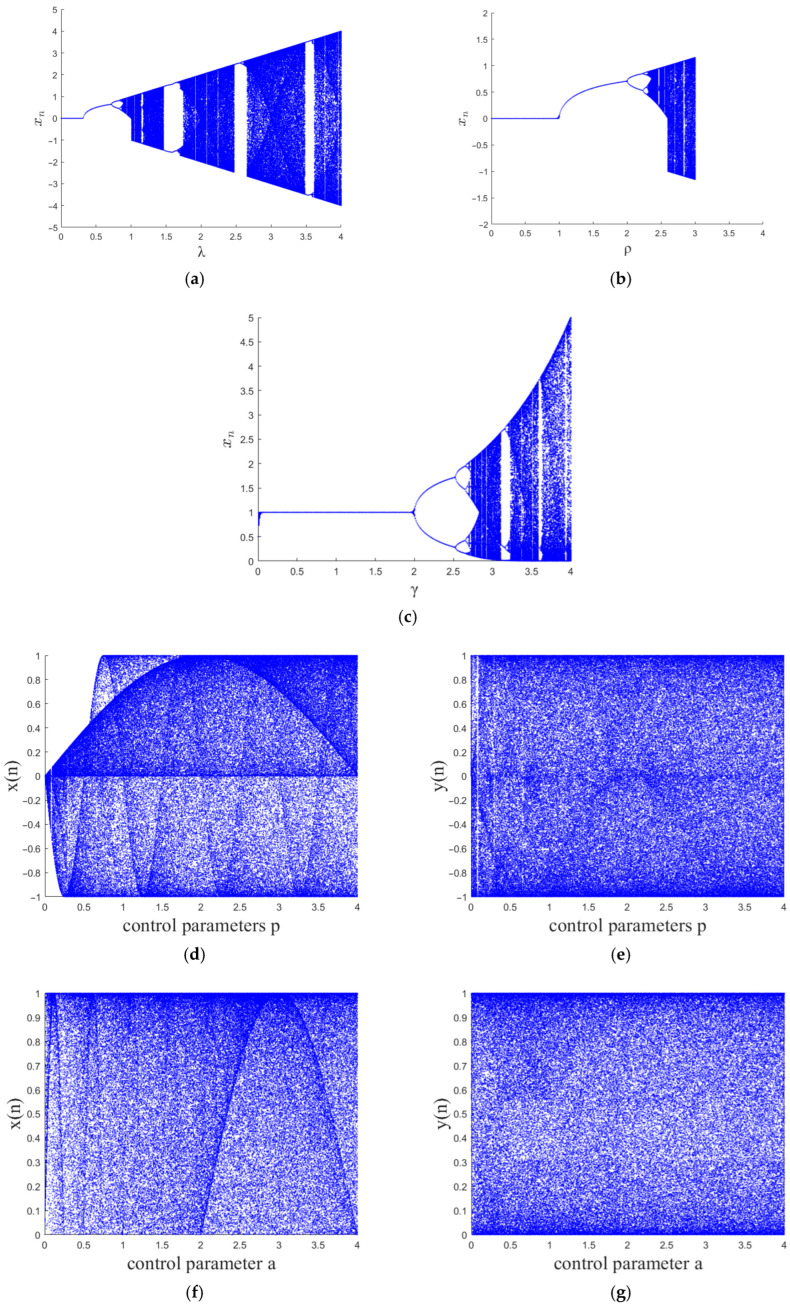

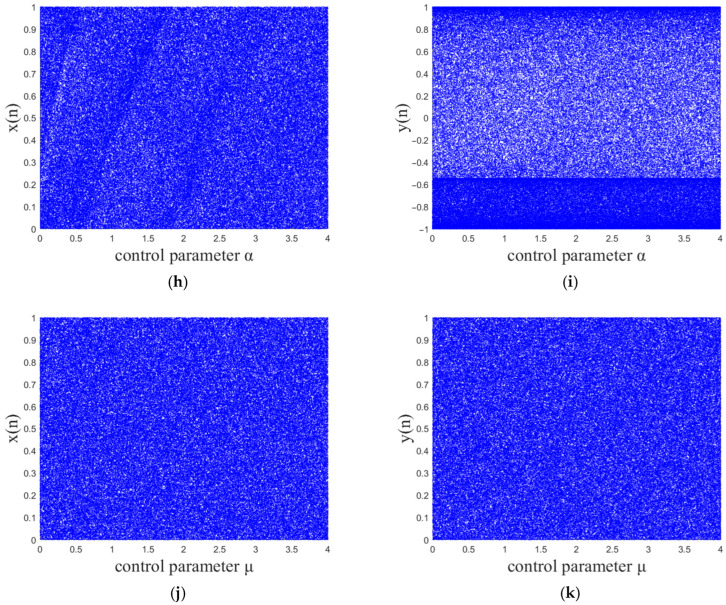
Bifurcation diagrams: (**a**) Sine map; (**b**) Cubic map; (**c**) May map; (**d**,**e**) 2D-CLSS; (**f**,**g**) 2D-CLII; (**h**,**i**) 2D-ILM; (**j**,**k**) 2D-SCMH.

Bifurcation diagrams visualize the evolution of the state of a system by depicting the relationship of state variables in a chaotic system as they vary with the control parameters. Figure 1 presents the bifurcation diagrams of each chaotic mapping. From Figure 1a–c, it can be observed that several classic chaotic maps exhibit periodic windows within the given parameter range. Figure 1d–k provides bifurcation diagrams of different chaotic maps. It can be observed that 2D-SCMH demonstrates a uniform distribution within the given parameter range, exhibits no periodic windows, and possesses a broader chaotic region and more complex dynamical characteristics.

#### 2.3.2. LE

**Figure 2:**
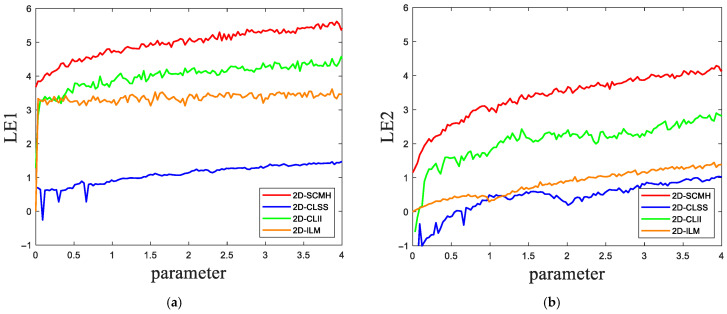
Comparison results of LE: (**a**) LE1; (**b**) LE2.

LE is the average exponential divergence between neighboring points in phase space and it is used to identify the numerical characteristics of chaotic motion. It is defined by Equation (5) [30]:

(5) $$LE=\underset{n\to \infty }{\lim}\frac{1}{n}\sum_{i=0}^{n-1}\ln\left|f^{\prime }(x_{i})\right|,$$

where *f* (*x_i_*) is a chaotic system. When the LE is positive, the system exhibits chaotic behavior, which is generally positively correlated with the magnitude of the LE value. If a 2D chaotic map has two LEs, and both LEs are greater than zero, the chaotic map is considered to be a hyperchaotic mapping. Hyperchaotic maps generally possess more complex and richer dynamical properties. Figure 2 illustrates the LE of different chaotic maps, where LE1 is the LE of output *X*, and LE2 is the LE of output *Y*. As clearly demonstrated in the figure, the LE of 2D-SCMH remains consistently greater than zero across the continuous parameter range, which confirms that this chaotic system is hyperchaotic. Moreover, within the parameter range, the LE of 2D-SCMH exceeds those of the other three 2D chaotic maps. Therefore, the 2D-SCMH exhibits more intricate chaotic behavior.

#### 2.3.3. Shannon Entropy

**Figure 3:**
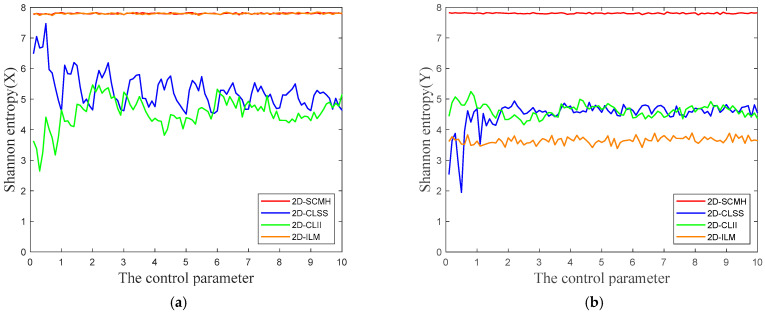
Comparison results for Shannon entropy: (**a**) Shannon entropy (*X*); (**b**) Shannon entropy (*Y*).

Shannon entropy is used to measure the uncertainty of a random variable. A higher entropy value indicates greater uncertainty in the random variable, making it more difficult to predict. It is defined by Equation (6) [31]:

(6) $$H(x)=-\sum_{i=1}^{n}p(x_{i})\log_{2}p(x_{i}),$$

where *x_i_* represents the *i*-th value of the chaotic sequence, and *p*(*x_i_*) denotes the probability of *x_i_*. For grayscale images, *n* = 256, so the maximum value of information entropy is log_2_(256) = 8. Figure 3 presents a comparison of Shannon entropy for different chaotic maps. From the figure, it can be seen that the Shannon entropy value of 2D-SCMH is closer to 8 than that of 2D-CLSS and 2D-CLII. Although the information entropy of the 2D-ILM output *X* is close to that of 2D-SCMH, the information entropy of the 2D-SCMH output *Y* is significantly closer to 8. Therefore, overall, the Shannon entropy value of 2D-SCMH is the closest to 8, demonstrating its randomness and unpredictability.

#### 2.3.4. Sample Entropy

**Figure 4:**
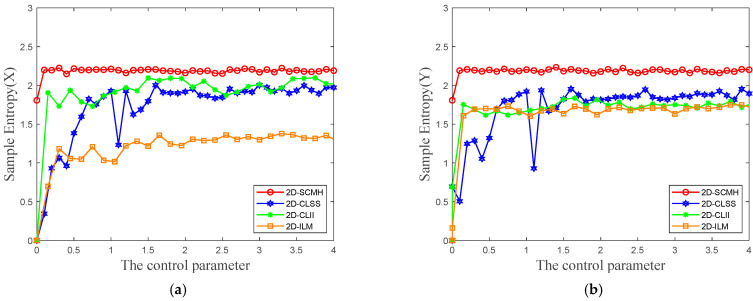
Comparison results for sample entropy: (**a**) sample entropy (*X*); (**b**) sample entropy (*Y*).

Sample entropy is a statistical measure used to quantify the complexity and regularity of time series. A smaller sample entropy value indicates higher self-similarity in the sequence, making it more predictable, while a larger sample entropy value suggests greater complexity and unpredictability of the sequence. Figure 4 illustrates the comparison of sample entropy among different chaotic maps. Across the entire parameter range, the sample entropy of 2D-SCMH remains consistently higher than that of the other maps, demonstrating that the sequences generated by 2D-SCMH possess higher complexity and stronger randomness.

#### 2.3.5. Sensitivity Analyses

Sensitivity analysis can be divided into initial value sensitivity analysis and control parameter sensitivity analysis. A well-performing chaotic system should exhibit high sensitivity to both initial values and control parameters, meaning that even tiny changes in the initial values or control parameters will lead to significant differences in the system output results.

**Figure 5:**
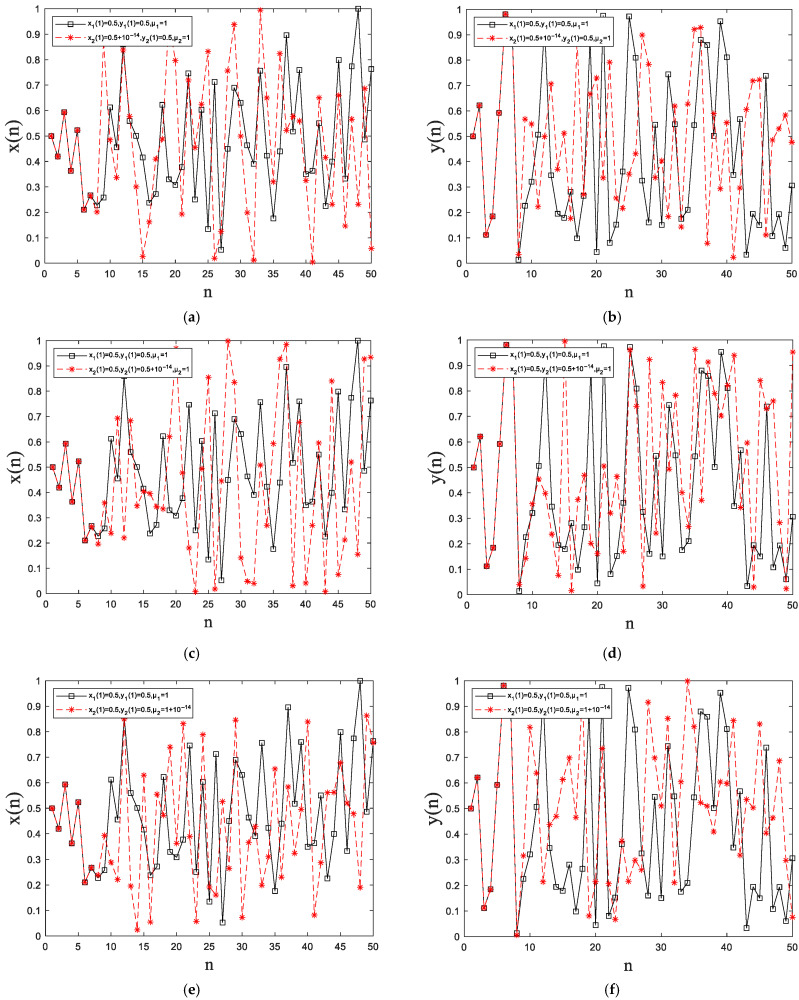
Sensitivity analyses: (**a,b**) minor changes in the initial values *x*_0_; (**c**,**d**) minor changes in the initial values *y*_0_; (**e**,**f**) minor changes in the control parameters *μ*.

Keeping *y*_1_(1) = 0.5, *y*_2_(1) = 0.5, *μ*_1_ = 1 and *μ*_2_ = 1 the same, let the initial values *x*_1_(1) = 0.5, *x*_2_(1) = 0.5 + 10^−14^. The difference between these two initial values is very small. Use 2D-SCMH for 50 iterations of these two initial values, respectively. Figure 5a,b show the output for *X* and *Y*, respectively, where the black line is the result of *x*_1_(1) iteration, and the red line is the result of *x*_2_(1) iteration. Similarly, keeping *x*_1_(1) = 0.5, *x*_2_(1) = 0.5, *μ*_1_ = 1 and *μ*_2_ = 1 the same, let *y*_1_(1) = 0.5, *y*_2_(1) = 0.5 + 10^−14^. Use 2D-SCMH for 50 iterations, respectively. Figure 5c,d show the output for *X* and *Y*, respectively. It can be seen that the black and red lines are not overlapping, which indicates that 2D-ICIM is highly sensitive to the initial values and *X* and *Y* interfere with each other. Finally, keeping *x*_1_(1) = 0.5, *x*_2_(1) = 0.5, *y*_1_(1) = 0.5 and *y*_2_(1) = 0.5 the same, let the control parameters *μ*_1_ = 1, *μ*_2_ = 1 + 10^−14^, respectively. Iterate 50 times with 2D-SCMH, respectively. Figure 5e,f show the output of *X* and *Y*, respectively. It can be seen that the red and black lines also do not overlap, which indicates that the 2D-ICIM has strong sensitivity to the control parameters as well.

#### 2.3.6. 0-1 Test

The 0-1 test is a method used to detect whether a dynamical system exhibits chaotic behavior. It calculates a statistical quantity *K* to determine whether the system behavior is chaotic or regular. A value of *K* approaching 1 indicates that the system is chaotic, while a value approaching 0 suggests that the system is regular. The test value *K* is calculated as follows [32]:

(7) $$K(c)=corr(\xi ,\Delta )=\frac{\mathrm{cov}(\xi ,\Delta )}{\sqrt{\mathrm{var}(\xi )\mathrm{var}(\Delta )}}\in [-1,1]$$

(8) $$\mathrm{cov}(x,y)=\frac{1}{q}\sum_{i=1}^{q}\left(x(i\right)-\bar{x})(y(i)-\bar{y}),$$

where ξ=1, 2, …, n, $\Delta =D_{c}(1),\;D_{c}(2),\;\ldots ,\;D_{c}(n)$, $\bar{x}=\frac{1}{q}\sum_{i=1}^{q}x(i)$ and var(x)=cov(x,x).

(9) $$D_{c}(n)=\underset{n\to \infty }{\lim}\frac{1}{N}\sum_{i=1}^{N}({\left(p_{c}(i+n)-p_{c}(i)\right)}^{2}+{\left(q_{c}(i+n)-q_{c}(i)\right)}^{2})-{\left(\underset{N\to \infty }{\lim}\frac{1}{N}\sum_{j=1}^{N}\phi (i)\right)}^{2}\frac{1-\cos(nc)}{1-\cos(c)},$$

where $p_{c}(n)=\sum_{i=1}^{n}\phi (i)\cos(ic),$ $q_{c}(n)=\sum_{i=1}^{n}\theta (i)\sin(ic)$, and c∈(0,π).

**Figure 6:**
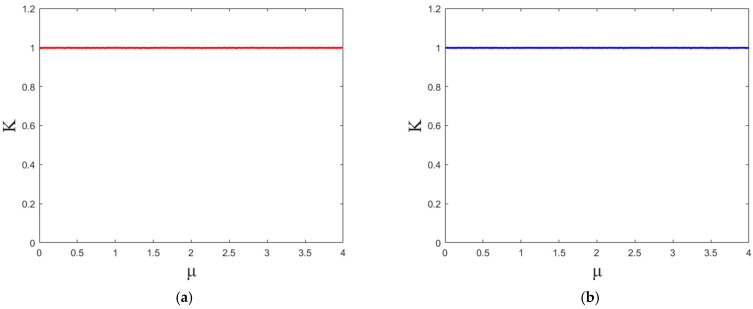
Results of the 0-1 test: (**a**) output *X* of the 2D-SCMH; (**b**) output *Y* of the 2D-SCMH.

Figure 6 presents the results of the 0-1 test. As can be observed, the *K* values for both outputs of the 2D-SCMH approach are close to 1, indicating that 2D-SCMH exhibits chaotic characteristics and irregular dynamical behavior.

#### 2.3.7. NIST Test

The randomness of the proposed 2D-SCMH was systematically evaluated using the 15 statistical test methods provided by the National Institute of Standards and Technology (NIST). If the *p*-value of each of the 15 subtests is greater than 0.01, it can be demonstrated that the chaotic sequence is random [33]. Table 1 presents the NIST test results, where *p*_1_ represents the test results for *X* and *p*_2_ denotes the test results for *Y*. It can be observed that each bit stream successfully passes all the tests. This demonstrates that the chaotic sequences generated by 2D-SCMH exhibit strong unpredictability.

## 3. Theoretical Principles

### 3.1. Dynamic Rhombus Transformation

The Manhattan distance, proposed by the 19th-century German mathematician Hermann Minkowski, is a metric used to measure the distance between two points in geometric space. It is defined as the sum of the absolute axial distances between two points in the 2D plane on the standard coordinate system. Specifically, given two points *A*(*x*_1_, *y*_1_) and *B*(*x*_2_, *y*_2_) in a 2D space, the Manhattan distance between them can be calculated by

(10) $$MD=|x_{1}-x_{2}|+|y_{1}-y_{2}|,$$

where *MD* is the Manhattan distance between point *A* and point *B*. After determining the transformation center point, each pixel in the image has a specific Manhattan distance *R* to this center point. For different values of *R*, connecting all pixels with the same *R* value forms multiple concentric rhombic structures, where the pixels along each rhombic boundary maintain an identical Manhattan distance from the center point.

**Figure 7:**
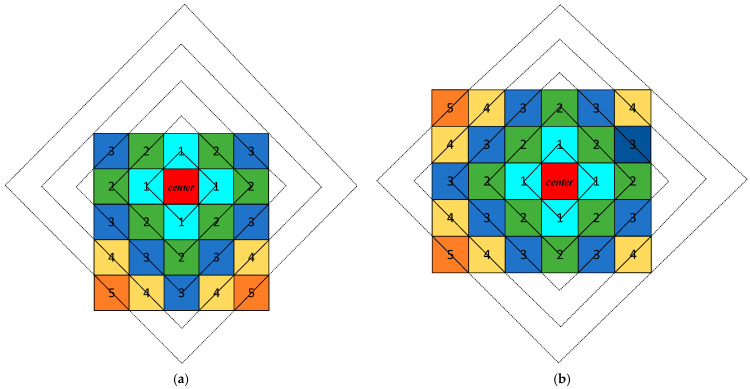
Dynamic rhombus transformation: (**a**) size of 5 × 5; (**b**) size of 5 × 6.

Using this property, we propose a dynamic rhombus transformation, as shown in Figure 7, where the number is the Manhattan distance from the pixel point to the transformation center. The solid and dashed lines denote scanned and unscanned elements, respectively. Different colors denote different sizes of rhombuses. Figure 7a shows the traversal process for a size 5 × 5 matrix with transformation center at (2, 3), and Figure 7b shows the traversal process for a size 5 × 6 matrix with transformation center at (3, 4). Therefore, the dynamic rhombus transformation can traverse images of any size using different transformation centers. Using chaotic sequences to dynamically select the transformation center and traversal order, we perform the dynamic rhombus transformation on images.

### 3.2. Digital Tube Model

**Figure 8:**
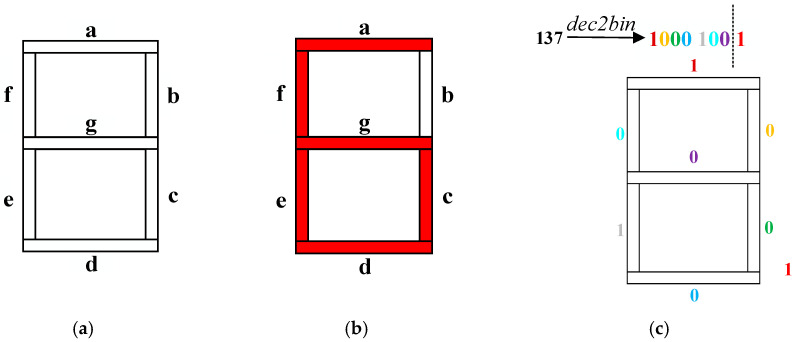
(**a**) Digital tube model; (**b**) example of the digital tube lighting up; (**c**) example of decimal number mapping to a digital tube.

A seven-segment digital tube is a common digital display device that consists of seven light-emitting diodes (LEDs) packaged together to form an “8” device. When different combinations of LEDs are illuminated, the digital tube can display different numbers between 0 and 9. Figure 8a is a digital tube model, where “*a*”–”*g*” represents different LEDs. For example, as shown in Figure 8b, when the LEDs represented by “*a*”, “*c*”, “*d*”, “*e*”, “*f*” and “*g*” represented by LEDs are lit, the digital tube will display the number “6”.

The designed digital tube model is described as follows. Since a decimal pixel value can be converted into 8 bits, the first 7 bits can be mapped to the digital tube model sequentially, with each LED representing one bit and the last bit handled separately.

Figure 8c shows an example of decimal mapping to a digital tube. In this example, the decimal number “137” is converted into “1000 1001” in binary, and the first 7 bits are mapped sequentially to the digital tube model, with the last bit handled separately.

## 4. Proposed Image Encryption Algorithm

### 4.1. Key Generation

To ensure that the generated key is associated with the plaintext image, the SHA-256 algorithm is employed to compute the hash value of the plain image. This hash value is then combined with the external key to generate the control parameters and initial values for 2D-SCMH.

**Step1:** Hash value generation

The SHA-256 algorithm is utilized to generate a 256-bit hash value *H* of the plaintext image *I* of size *m* × *n*. *H* is divided into 32 8-bit binary groups, and each group is converted into one decimal number, which can be expressed as *H* = (*h*_1_, *h*_2_, …, *h*_32_).

**Step2:** Key generation

The initial values and control parameters of 2D-SCMH can be calculated by

(11) $$\left\{\begin{array}{l} \mu =\frac{h_{1}⊕h_{3}}{\mathrm{mod}(e_{1},1)+2^{8}}+\frac{h_{29}⊕h_{31}}{\mathrm{mod}(e_{2},1)+2^{8}}\\ x_{0}=\frac{\left(h_{5}+h_{7})⊕(h_{9}+h_{11})⊕(h_{13}+h_{15}\right)}{512}\\ y_{0}=\frac{\left(h_{17}⊕h_{19})+(h_{21}⊕h_{23})+(h_{25}⊕h_{27}\right)}{512} \end{array}\right.,$$

(12) $$\left\{\begin{array}{l} \mu^{\prime }=\frac{h_{2}⊕h_{4}}{\mathrm{mod}(e_{3},1)+2^{8}}+\frac{h_{30}⊕h_{32}}{\mathrm{mod}(e_{4},1)+2^{8}}\\ {x_{0}}^{\prime }=\frac{\left(h_{6}+h_{8})⊕(h_{10}+h_{12})⊕(h_{14}+h_{16}\right)}{512}\\ {y_{0}}^{\prime }=\frac{\left(h_{18}⊕h_{20})+(h_{22}⊕h_{24})+(h_{26}⊕h_{28}\right)}{512} \end{array}\right.,$$

where ⊕ is the XOR operation, and *e*_1_, *e*_2_, *e*_3_ and *e*_4_ are the external keys.

**Step3:** Chaotic sequence generation

The control parameter *μ* and the initial values *x*_0_ and *y*_0_ of 2D-SCMH are used for *m* × *n* iterations to obtain two chaotic sequences *W* and *X* of the length *m* × *n*. The control parameter *μ*′ and the initial values *x*_0_′ and *y*_0_′ of 2D-SCMH are used for *m* × *n* iterations to obtain another two chaotic sequences *Y* and *Z* of the length *m* × *n*.

### 4.2. Encryption Process

**Figure 9:**
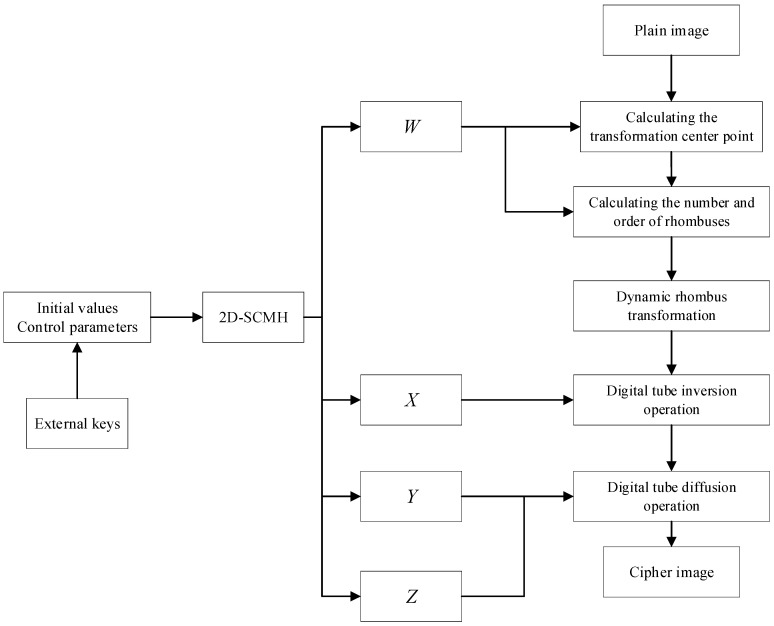
Flow chart of the proposed encryption process.

The encryption steps are described in detail, including the scrambling and diffusion stages. Figure 9 shows the flow chart of the proposed encryption algorithm. Algorithm 1 provides the pseudo-code for the encryption process.

**Step 1:** Calculating the transformation center point

The transformation center point should be selected for the dynamic rhombus transformation. The transformation center point (*C*_1_, *C*_2_) can be calculated by

(13) $$\left\{\begin{array}{l} C_{1}=\mathrm{mod}(floor(W(1))\times 10^{16}),m)+1\\ C_{2}=\mathrm{mod}(floor(W(2))\times 10^{16}),n)+1 \end{array}\right.,$$

where *floor*(·) is the negative infinity rounding function.

**Step 2:** Calculating the number of rhombuses

To ensure that all pixels in the image are traversed, it is necessary to determine the number of rhombuses to be traversed. The number of rhombuses should be determined by the pixel points at the furthest Manhattan distance from the transformation center point of the traversal to the image. If the pixel at the maximum Manhattan distance from the transformation center has been traversed, it indicates that the largest rhombus has extended to the image boundary, thereby ensuring that all pixels have been completely traversed. It can be confirmed that the pixel with the maximum Manhattan distance from the transformation center is certain to be among the four vertices of the image. The Manhattan distances *h*_1_, *h*_2_, *h*_3_ and *h*_4_ from the transformation center point to the pixel points in the upper-left corner (1, 1), upper-right corner (1, *n*), lower-left corner (*m*, 1), and lower-right corner (*m*, *n*) of *I* can be calculated by

(14) $$\left\{\begin{array}{l} h_{1}=abs(C_{1}-1)+abs(C_{2}-1)\\ h_{2}=abs(C_{1}-1)+abs(C_{2}-n)\\ h_{3}=abs(C_{1}-m)+abs(C_{2}-1)\\ h_{4}=abs(C_{1}-m)+abs(C_{2}-n) \end{array}\right.,$$

where *abs*(·) is the absolute value function.

Maximum Manhattan distance *L* can be calculated by

(15) $$L=max(h_{1},h_{2},h_{3},h_{4}),$$

where *max*(·) is the maximum function, and *L* is the number of transformation rhombuses.

**Step 3:** Calculating the traversal order of a rhombus

Skipping the first 2 values, sequentially take *L* elements from the chaotic sequence *W* and denote them by *W*′ as shown Equation (16). The traversal order *P* of the rhombuses can be calculated by Equation (17).

(16) $$W^{\prime }=W(3:L+2),$$

(17) $$[W^{\prime \prime },P]=sort(W^{\prime }),$$

where *sort*(·) is the ascending sorting function, *P* is the index sequence, and *W*″ is the new sequence after sorting.

**Step 4:** Dynamic rhombus transformation

**Figure 10:**
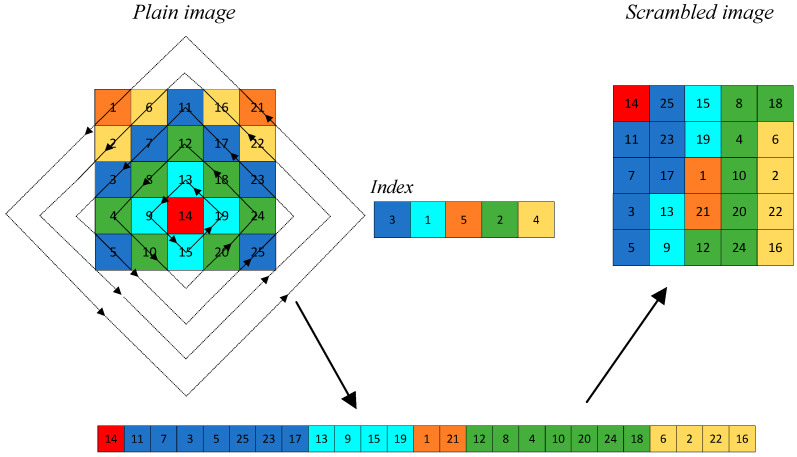
Dynamic rhombus transformation.

First, with (*C*_1_, *C*_2_) as the transformation center point, all pixel points on the *P*_1_-th rhombus outward from (*C*_1_, *C*_2_) are traversed sequentially to form a 1D sequence *R*_1_; second, all pixel points on the *P*_2_-th rhombus outward from (*C*_1_, *C*_2_) are traversed sequentially to form a 1D sequence *R*_2_; …; again, all pixels on the *P_L_*-th rhombus outward from (*C*_1_, *C*_2_) are traversed sequentially to form a 1D sequence *R_L_*, where {*P*_1_, *P*_2_, …, *P_L_*} ∈ *P*. While traversing each pixel point, it is necessary to determine whether the pixel point is an element of *I*. If it is, the pixel point is placed into the corresponding 1D sequence; otherwise, the pixel point is not included in the corresponding 1D sequence. Finally, the set {*R*_1_, *R*_2_, …, *R_L_*} is concatenated end-to-end to generate a 1D sequence *R*, *I*(*C*_1_, *C*_2_) is added to the beginning of *R*, and then *R* is converted into a 2D image E of size *m* × *n*, which is the final scrambled image. As an example, the transformation process of dynamic rhombus transformation with a 5 × 5 matrix size is shown in Figure 10, where the arrows indicate the direction of scanning and different colors denote different sizes of rhombuses.


**Algorithm 1: Encryption process**
**Input:** the plain image *I* and the chaotic sequences *W*, *X*, *Y* and *Z* generated in the Section 4.1 **Output:** the cipher image *C* 1: [*m*, *n*] = *size*(*I*) 2: /* Calculating the transformation center point */ 3: *C*_1_ ← *mod*(*floor*(*W*(1) × 10^16^), *m*) + 1 4: *C*_2_ ← *mod*(*floor*(*W*(2) × 10^16^), *n*) + 1 5: /* Calculating the number and order of rhombuses */ 6: *h*_1_ ← *abs*(*C*_1_-1)+*abs*(*C*_2_-1) 7: *h*_2_ ← *abs*(*C*_1_-1)+*abs*(*C*_2_-*n*) 8: *h*_3_ ← *abs*(*C*_1_-*m*)+*abs*(*C*_2_-1) 9: *h*_4_ ← *abs*(*C*_1_-*m*)+*abs*(*C*_2_-*n*) 10: *L* ← *max*(*h*_1_, *h*_2_, *h*_3_, *h*_4_) 11: *W*′ ← *W*(3: *L+*2) 12: *P* ← *sort*(*W*′) 13: /* Dynamic rhombus transformation */ 14: **for**
*t* ← 1 to *L*
**do** 15:    *R*(*t*) ← [ ] 16:    **for**
*i* ← 1 to *m*
**do** 17:       **for**
*j* ← 1 to *n*
**do** 18:          *MD*←*abs*(*i*-*C*_1_)+ *abs*(*j*-*C*_2_) 19:          **if**
*MD*=*P*(*t*) **then** 20:             *R*(*t*) ← (*R*(*t*), *I*(*i*, *j*)) 21:          **end** 22:       **end** 23:    **end** 24: **end** 25: *result* ← [ ] 26: **for**
*i* ← 1 to *L*
**do** 27:      *result* ← [*result*, *R*(*i*)] 28: **end** 29: *result* ← (*I*(*C*_1_, *C*_2_), *result*) 30: *E* ← *reshape*(*result*, *m*, *n*) 31: /* Normalizing chaotic sequences */ 32: *S*_1_ ← *mod*(*floor*(*X* × 10^16^), 256) 33: *S*_2_ ← *mod*(*floor*(*Y* × 10^16^), 10) 34: *S*_3_ ← *mod*(*floor*(*Z* × 10^16^), 7) 35:/* Digital tube inversion and diffusion operation */ 36: *E* ← *reshape*(*E*, 1, *m* × *n*) 37: **parfor**
*i* ← 1 to *m* × *n*
**do** 38:    **switch**
*S*_2_(*i*) **do** 39:          **case** 0 40:              *V* ← [1, 2, 3, 4, 5, 6] 41:          **case** 1 42:              *V* ← [2, 3] 43:          **case** 2 44:              *V* ← [1, 2, 4, 5, 7] 45:          **case** 3 46:              *V* ← [1, 2, 3, 4, 7] 47:          **case** 4 48:              *V* ← [2, 3, 6, 7] 49:          **case** 5 50:              *V* ← [1, 3, 4, 6, 7] 51:          **case** 6 52:              *V* ← [1, 3, 4, 5, 6, 7] 53:          **case** 7 54:              *V* ← [1, 2, 6] 55:          **case** 8 56:              *V* ← [1, 2, 3, 4, 5, 6, 7] 57:          **case** 9 58:              *V* ← [1, 2, 3, 4, 6, 7] 59:          **endsw** 60:    **endsw** 61:       *e* ← *dec2base*(*E*(*i*), 2, 8) 62:       **for**
*j* ← 1 to *length*(*V*) **do** 63:              *temp* ← *e* (*V*(*j*)) 64:              **if**
*temp* = ‘0’ **then** 65:                  *modified_bit* ← ‘1’ 66:              **else** 67:                  *modified_bit* ← ‘0’ 68:              **end** 69:            *e*(*V*(*j*)) ← *modified_bit* 70:       **end** 71:     *s*_1_ ← *dec2base*(*S*_1_(*i*), 2, 8) 72:     *temp* ← *s*_1_(1:7) 73:     *temp*′ ← *circshift*(*temp*, *S*_3_(*i*)) 74:     *s*_1_′ ← (*temp*′, *s*_1_(8)) 75: *K*(*i*) ← *bin2dec*(*s*_1_′**⊕***e*) 76: **end** 77: *C* ← *reshape*(*K*, *m*, *n*) Remark: Notations are represented by ‘/* */’. 

**Step 5:** Normalizing chaotic sequences

The chaotic sequences *X*, *Y* and *Z* are normalized into *S*_1_ to *S*_3_ by

(18) $$\left\{\begin{array}{l} S_{1}=\mathrm{mod}(floor(X\times 10^{16}),256)\\ S_{2}=\mathrm{mod}(floor(Y\times 10^{16}),10)\\ S_{3}=\mathrm{mod}(floor(Z\times 10^{16}),7) \end{array}\right..$$

**Step 6:** Digital tube inversion operation

The pixel value of scrambled image *E* is converted into 8 bits in binary, and the first 7 bits are mapped to the digital tube model sequentially. *S*_2_ is utilized to select the number displayed on the digital tube and invert the binary bits corresponding to the corresponding light-emitting diode combinations, where “0” is changed to “1” and “1” is changed to “0”. Additionally, the last bit is left out of the operation for the time being.

**Step 7:** Digital tube diffusion operation

The chaotic sequence *S*_1_ is converted to 8-bit binary. The first 7 bits are sequentially mapped to the digital tube model, which is circularly shifted according to the digital tube model, with the number of shifted bits controlled by *S*_3_. After that, a bit-by-bit XOR operation is performed with the inverted scrambled image in accordance with the corresponding binary bits of the digital tube model. The last bit of the pixel value of *E* and the last bit of the pixel value of *S*_1_ are XORed separately. Finally, the result of the XOR operation is concatenated with the result of the last bit operation and converted to decimal, which is the diffusion result. The digital tube inversion and diffusion operations are applied to each pixel of *E*, generating the final cipher image *C*. Because the digital tube inversion operation and digital tube diffusion operation are executed independently for each pixel, the pixels do not interfere with each other, the encryption can be accelerated by parallel processing.

**Figure 11:**
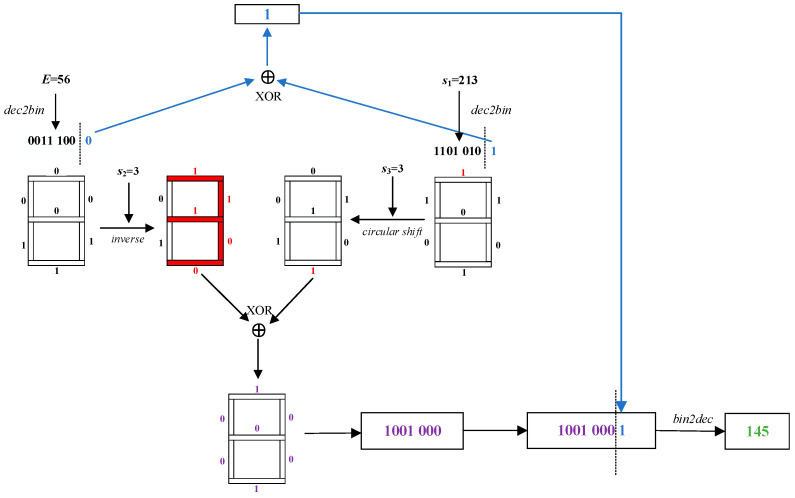
Dynamic diffusion algorithm based on digital tube model.

The schematic diagram of the dynamic diffusion algorithm based on the digital tube model is illustrated in Figure 11. Take *E* = 56, chaotic sequences *s*_1_ = 213, *s*_2_ = 3 and *s*_3_ = 3 as examples. In this case, the pixel value 56 of *E* and the chaotic value 213 of *s*_1_ are each converted to 8-bit binary, and their respective first 7 bits are sequentially mapped onto the digital tube model. Owing to *s*_2_ = 3, the digital tube model accordingly displays the numeral “3”, and the corresponding bits of pixel value 56 of *E* are inverted. Owing to *s*_3_ = 3, the first 7 bits of the *s*_1_ value “213” are circularly shifted right by 3 bits along the digital tube model. After that, the bit-by-bit XOR is performed according to the digital tube model, and the last bit is XORed individually. Finally, the result of the XOR is spliced and converted to decimal, which is the final diffusion result value “145”.

### 4.3. Decryption Process

**Figure 12:**
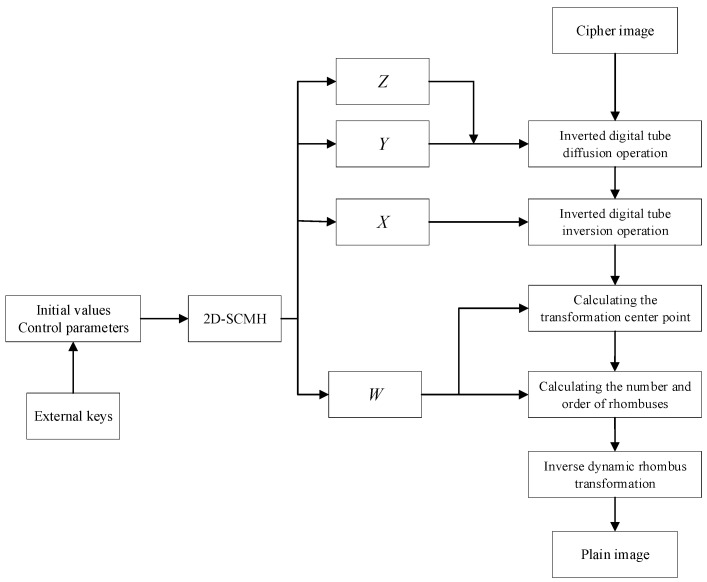
Flow chart of the proposed decryption algorithm.

The decryption steps are described in detail. The image decryption process is the inverse of the image encryption process and its flow chart is shown in Figure 12. Algorithm 2 is the pseudo-code of the decryption process.

**Step 1:** Chaotic sequence generation

The received keys (*μ*, *x*_0_, *y*_0_) are iterated through the 2D-SCMH *m × n* times to generate chaotic sequences *W* and *X* of length *m* × *n*. Similarly, the received keys (*μ*′, *x*_0_′, *y*_0_′) are iterated through 2D-SCMH *m* × *n* times to generate chaotic sequences *Y* and *Z* of length *m* × *n*. The chaotic sequence *X*, *Y*, and *Z* is normalized by Equation (18) to obtain the sequence *S*_1_, *S*_2_ and *S*_3_.

**Step 2:** Inverted digital tube diffusion operation

Map the first 7 bits of the pixel value of sequence *S*_1_ to the digital tube model for inverse circular shifting. The number of bits of the inverse circular shifting is controlled by *S*_3_. Subsequently, the first 7 bits of the pixel value of cipher image *C* are also mapped onto the digital tube model. A bit-by-bit XOR operation is performed with the inversely circularly shifted *S*_1_ according to the digital tube model to obtain the elements prior to the digital tube diffusion operation. Additionally, the last bit of the pixel value of *C* and the last bit of the pixel value of *S*_1_ are individually subjected to the XOR operation.

**Step 3:** Inverted digital tube inversion operation

The result obtained from Step 2 is utilized to select the digits displayed on the digital tube using *S*_2_. The binary bits corresponding to the respective light-emitting diode combinations are inverted, where the last bit is left out of the operation. The inverted result is concatenated with the last bit and converted to decimal, yielding the final computation result. Finally, the inverted digital tube diffusion operation and inverted digital tube inversion operation are applied to each pixel of the cipher image *C* to obtain the scrambled image *E*. Since the individual pixels are non-interfering with each other, the encryption can also be accelerated by parallel processing.

**Step 4:** Calculating the transformation center point

The transformation center point (*C*_1_, *C*_2_) can be calculated by Equation (13).

**Step 5:** Calculating the number and order of rhombuses

Calculate the number of rhombuses *L* by Equations (14) and (15), and the transformation order of a rhombus *P* by Equations (16) and (17).

**Step 6:** Inverse dynamic rhombus transformation

At the beginning of the inverse dynamic rhombus transformation process, a recovery matrix *Q* is constructed with the same size as the scrambled image *E*, and all pixel values are initialized to 0. Convert the scrambled image *E* into a 1D sequence *R*, where the first value of *R* is the transformation center point (*C*_1_, *C*_2_) of the recovery matrix. Replace the zero-value element at the position (*C*_1_, *C*_2_) in *Q* with this value and remove it from *R*. Traverse all pixel points on the *P*_1_-th rhombus of *Q*, sequentially replace the zero-value elements in *Q* with the current elements from *R*, and remove the replaced elements from *R*; traverse all pixel points on the *P*_2_-th rhombus of *Q*, sequentially replace the zero-value elements in *Q* with the current elements from *R*, and remove the replaced elements from *R*; …; traverse all pixel points on the *P_L_*-th rhombus *Q*, sequentially replace the zero-value elements in *Q* with the current elements from *R*, and remove the replaced elements from *R*. During the traversal of each pixel point, it is necessary to determine whether the zero-value element belongs to *Q*. If it does, replace it with the corresponding element from *R*, and remove the replaced element from *R*; otherwise, no replacement is performed. Finally, after all the elements in *R* have been replaced, *Q* is the plain image *I*.


**Algorithm 2: Decryption process**
**Input:** the cipher image *C* and the chaotic sequences *W*, *X*, *Y* and *Z* generated in Section 4.1 **Output:** the plain image *I* 1: [*m*, *n*] = *size*(*C*) 2: /* Chaotic sequence generation */ 3: *S*_1_ ← *mod*(*floo*r(*X* × 10^16^), 256) 4: *S*_2_ ← *mod*(*floo*r(*Y* × 10^16^), 10) 5: *S*_3_ ← *mod*(*floo*r(*Z* × 10^16^), 7) 6: /* Inversed digital tube inversion and diffusion operation */ 7: *C* ← *reshape*(*C*, 1, *m* × *n*) 8: **parfor**
*i←*1 to *m* × *n*
**do**
9:      *c* ← *dec2base*(*C*(*i*), 2, 8) 10:    *s*_1_ ← *dec2base*(*S*_1_(*i*), 2, 8) 11:    *temp* ← *s*_1_(1:7) 12:    *temp*′ ← *circshift*(*temp*, -*S*_3_(*i*)) 13:    *s*_1_′ ← (*temp*′, *s*_1_(8)) 14:    *k* ← *c***⊕***s*_1_′ 15:    **switch**
*S*_2_(*i*) **do** 16:          **case** 0 17:              *V* ← [1, 2, 3, 4, 5, 6] 18:          **case 1** 19:              *V* ← [2, 3] 20:          **case** 2 21:              *V* ← [1, 2, 4, 5, 7] 22:          **case** 3 23:              *V* ← [1, 2, 3, 4, 7] 24:          **case** 4 25:              *V* ← [2, 3, 6, 7] 26:          **case** 5 27:              *V* ← [1, 3, 4, 6, 7] 28:          **case** 6 29:              *V* ← [1, 3, 4, 5, 6, 7] 30:          **case** 7 31:              *V* ← [1, 2, 6] 32:          **case** 8 33:              *V* ← [1, 2, 3, 4, 5, 6, 7] 34:          **case** 9 35:              *V* ← [1, 2, 3, 4, 6, 7] 36:          **endsw** 37:    **endsw** 38:    **for**
*j* ←1 to *length*(*V*) **do** 39:                *temp* ← *k* (*V*(*j*)) 40:                **if**
*temp* = ‘0’ **then** 41:                    *modified_bit* ← ‘1’ 42:                **else** 43:                    *modified_bit* ← ‘0’ 44:                **end** 45:              *k*(*V*(*j*)) ← *modified_bit* 46:       **end** 47:       *e*(*i*) ← *bin2dec*(*k*) 48:    **end** 49: *E* ← *reshape*(*e*, *m*, *n*) 50: /* Calculating the transformation center point */ 51: *C*_1_ ← *mod*(*floor*(*W*(1) × 10^16^), *m*) + 1 52: *C*_2_ ← *mod*(*floor*(*W*(2) × 10^16^), *n*) + 1 53: /* Calculating the number and order of rhombuses */ 54: *h*_1_ ← *abs*(*C*_1_-1) + *abs*(*C*_2_-1) 55: *h*_2_ ← *abs*(*C*_1_-1) + *abs*(*C*_2_-*n*) 56: *h*_3_ ← *abs*(*C*_1_-*m*) + *abs*(*C*_2_-1) 57: *h*_4_ ← *abs*(*C*_1_-*m*) + *abs*(*C*_2_-*n*) 58: *L* ← *max*(*h*_1_, *h*_2_, *h*_3_, *h*_4_) 59: *W*′ ← *W*(3: *L +* 2) 60: *P* ← *sort*(*W*′) 61:/* Inverse dynamic rhombus transformation */ 62: *Q* ← *zeros*(*m*, *n*) 63: *R* ← *reshape*(*E*, 1, *m* × *n*) 64: *Q*(*C*_1_, *C*_2_) ← *R*(1) 65: *R*(1) ← [ ] 66: **for**
*t* ← 1 to *L*
**do** 67:    **for**
*i* ← 1 to *m*
**do** 68:       **for**
*j* ← 1 to *n*
**do** 69:          *MD* ← *abs*(*i*-*C*_1_) + *abs*(*j*-*C*_2_) 70:          **if**
*MD* = *P*(*t*) **then** 71:             Q(*i*, *j*) ← *R*(1) 72:              *R*(1) ← [ ] 73:          **end** 74:       **end** 75:    **end** 76: **end** 77: *I* ← *Q*

## 5. Experimental Results

**Figure 13:**
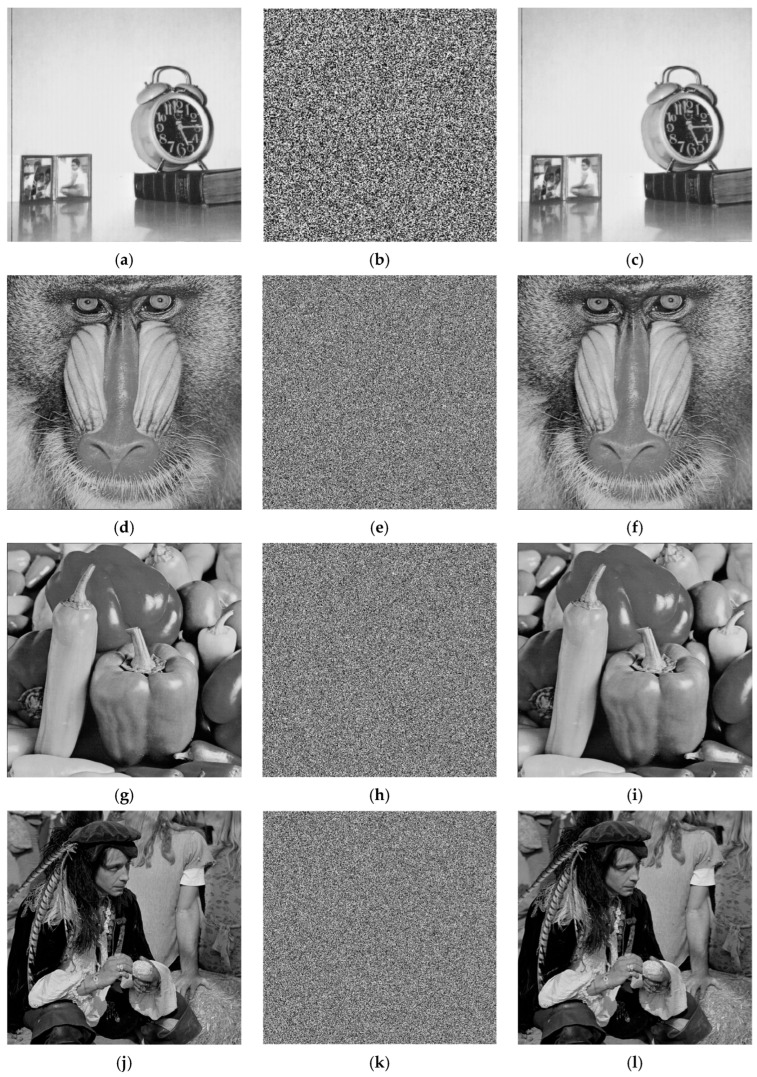
Experimental results: (**a**,**d**,**g**,**j**): plain images; (**b**,**e**,**h**,**k**): cipher images; (**c**,**f**,**i**,**l**): decrypted images. (Top to down: Clock (256 × 256), Baboon (512 × 512), Peppers (512 × 512) and Male (1024 × 1024)).

MATLAB R2023b was used as a simulation platform to evaluate our proposed algorithms. The experiments were performed on a computer with 14th generation Intel^®^ Core™ i9-14900HX processor (2.20 GHz) and 16 GB of RAM. All the grayscale images (Clock, Baboon, Peppers and Male) used in our experiment are from the USC-SIPI image database (http://sipi.usc.edu/database) accessed on 13 October 2024. The image sizes are as follows: Clock is 256 × 256, Baboon and Peppers are 512 × 512, and Male is 1024 × 1024. The external keys are set to *e*_1_ = 0.7586, *e*_2_ = 0.4152, *e*_3_ = 0.7482 and *e*_4_ = 0.6152. Figure 13 illustrates the experimental results. As can be observed from Figure 13, the images are encrypted into unrecognizable noise-like patterns, effectively concealing the information of the plain images. Moreover, the decrypted images exhibit no visual differences compared to the plain images.

## 6. Security Analyses

To evaluate the security and efficiency of the proposed algorithm, a series of tests were conducted, including histogram analysis, key space analysis, key sensitivity analysis, correlation analysis, information entropy analysis, differential attack analysis, cropping attack, noise attack and encryption speed analysis. Meanwhile, the results were compared with those of similar existing algorithms.

### 6.1. Histogram Analysis

**Figure 14:**
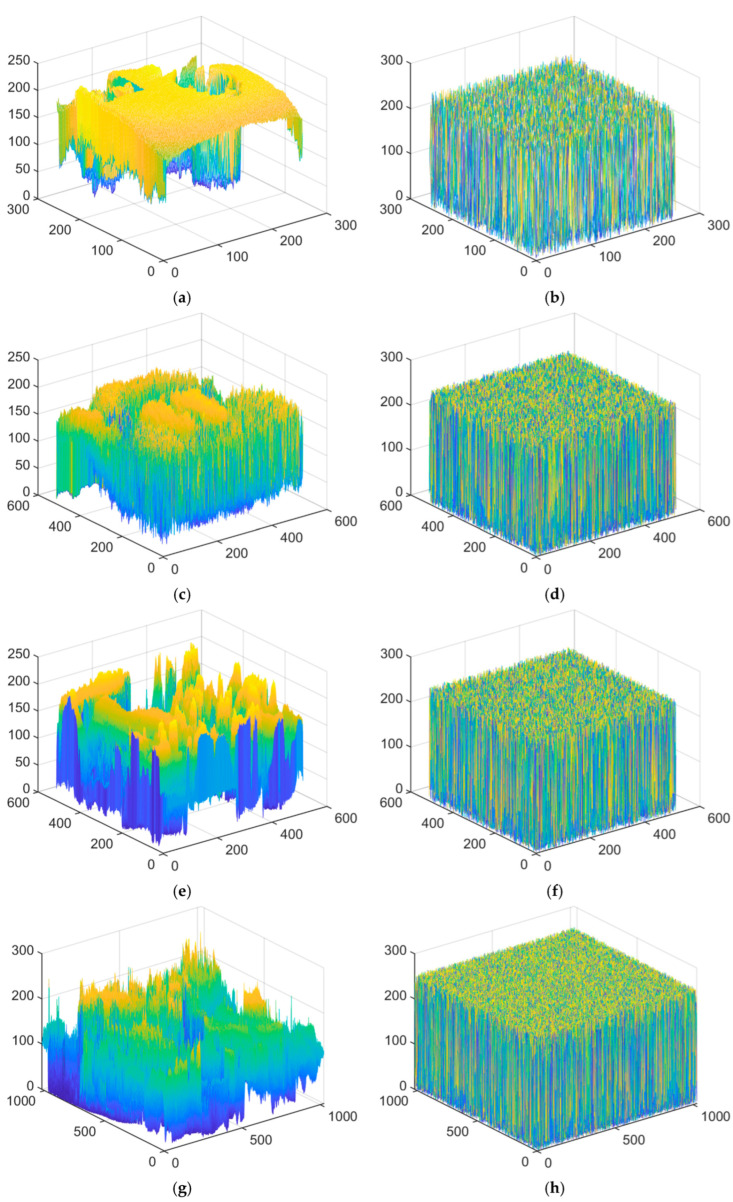
Histogram analysis: (**a**,**c**,**e**,**g**): plain images, (**b**,**d**,**f**,**h**): cipher images. (Top to down: Clock (256 × 256), Baboon (512 × 512), Peppers (512 × 512) and Male (1024 × 1024)).

The histogram depicts the distribution of grayscale values in an image, providing a visual representation of the image’s statistical characteristics. Figure 14 illustrates the histograms of both the plain image and the encrypted image, where the colors are automatically generated based on the grayscale values of the images for visualization purposes. As can be observed from the figure, the pixel value distribution of the cipher image approaches a uniform distribution. This indicates that the proposed encryption algorithm effectively conceals the information of the plain image.

To further verify the uniformity of pixel distribution in the ciphertext image, a chi-square test is employed to evaluate the encrypted image. $\chi^{2}\;$ can be calculated by Equation (19) [34]:

(19) $$\chi^{2}=\sum_{k=0}^{255}\frac{{\left(p_{k}-p\right)}^{2}}{p},$$

where *p_k_* presents the frequency of pixels with a gray value of *i*, and *p* denotes the expected quantity of pixel *k.* When the significance level is 0.05, $\chi_{0.05}^{2}$(255) = 293.24783. If the calculated $\chi^{2}$ statistic of the cipher image is less than 293.24783, it can be concluded that the ciphertext image passes the $\chi^{2}$ statistical test. Table 2 presents the results of the $\chi^{2}$ test. As can be observed from the table, all test images passed the test, indicating that the proposed encryption algorithm exhibits strong resistance against the statistical analysis attack.

### 6.2. Key Space Analysis

The key space is the set of all possible keys. In general, a secure image encryption algorithm requires that the key space is larger than 2^100^ to resist exhaustive brute force attacks [35]. In the proposed image encryption algorithm, the key space of the chaotic system consists of initial values *x*_0_, *y*_0_, *x*_0_′, *y*_0_′ and control parameters *μ*, *μ*′. If the computational accuracy of the computer is 10^−14^, the key space is (10^14^)^6^ = 10^84^ ≈ 2^279^ >> 2^100^. Therefore, the proposed encryption algorithm has a large enough key space to resist the exhaustive attack.

### 6.3. Key Sensitivity Analysis

**Figure 15:**
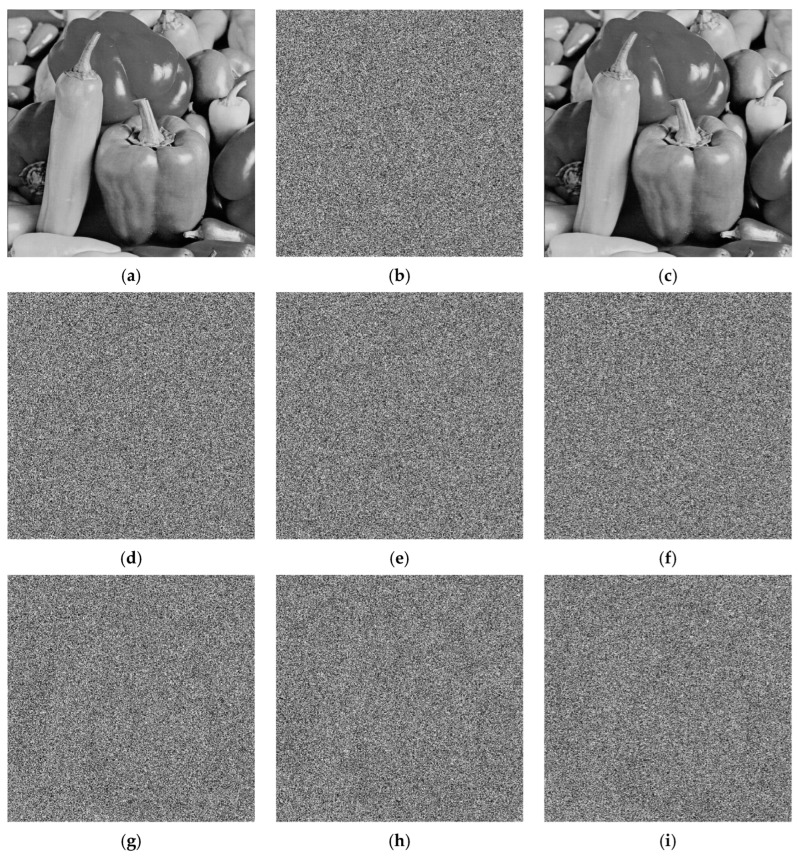
Key sensitivity analysis results: (**a**) Peppers plain image; (**b**) Peppers cipher image; (**c**) the correct key; (**d**–**i**) the incorrect keys.

An encryption algorithm with strong key sensitivity can produce significantly different encrypted images when the encryption key undergoes even a minor change. Assuming the correct key is *key*_0_ = {*μ*, *x*_0_, *y*_0_, *μ*′, *x*_0_′, *y*_0_′}, we alter *μ*′ by 10^−14^ to obtain an incorrect key *key*_1_ = {*μ* + 10^−14^, *x*_0_, *y*_0_, *μ*′, *x*_0_′, *y*_0_′}. Similarly, incorrect *key*_2_ = {*μ*, *x*_0_ + 10^−14^, *y*_0_, *μ*′, *x*_0_′, *y*_0_′}, *key*_3_ = {*μ*, *x*_0_, *y*_0_ + 10^−14^, *μ*′, *x*_0_′, *y*_0_′}, *key*_4_ = {*μ*, *x*_0_, *y*_0_, *μ*′ + 10^−14^, *x*_0_′, *y*_0_′}, *key*_5_ = {*μ*, *x*_0_, *y*_0_, *μ*′, *x*_0_′ + 10^−14^, *y*_0_′} and *key*_6_ = {*μ*, *x*_0_, *y*_0_, *μ*′, *x*_0_′, *y*_0_′ + 10^−14^} are generated. Use these keys to decrypt the cipher image in order. Figure 15 shows the results of the key sensitivity analysis, and it can be seen that only the correct key can fully recover the original image. This indicates that the proposed encryption algorithm exhibits strong key sensitivity.

### 6.4. Correlation Analysis

**Figure 16:**
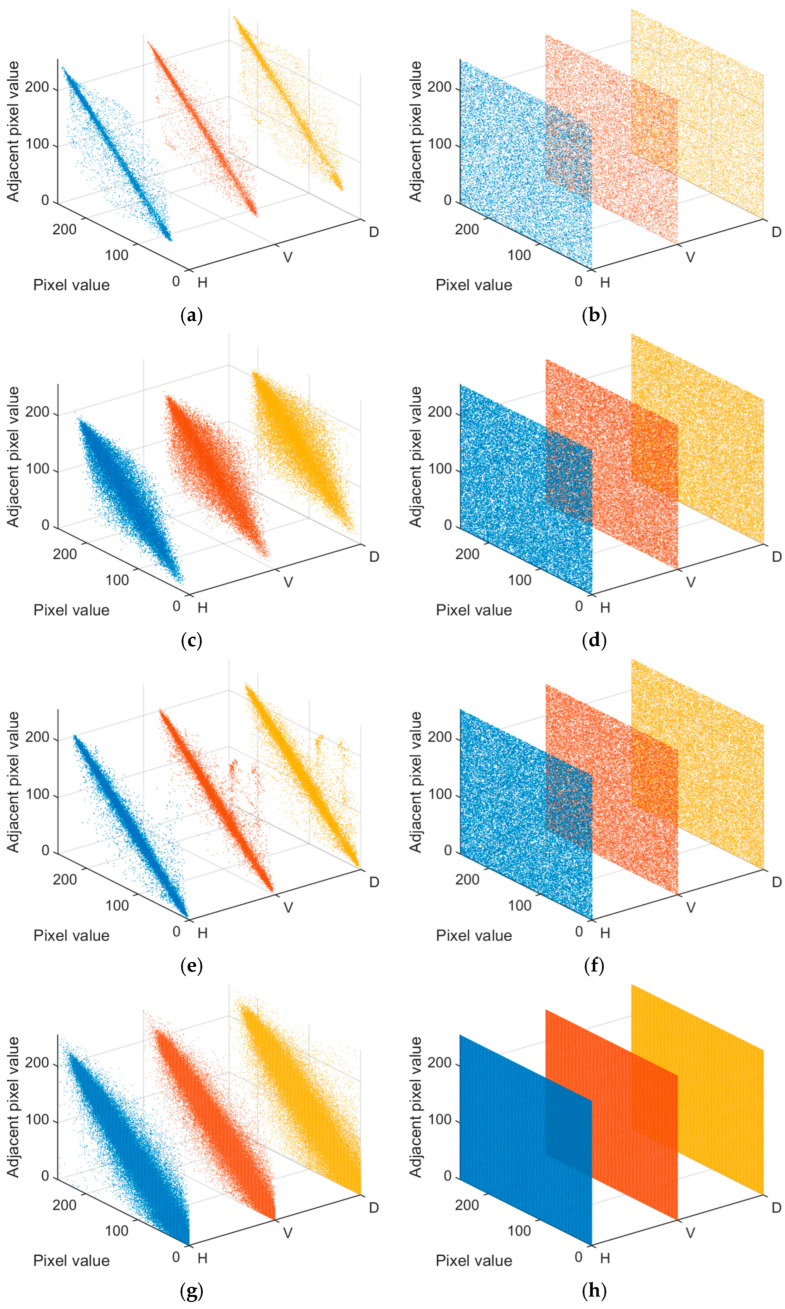
Correlation distributions of the plain images and their cipher images: (**a**,**c**,**e**,**g**): plain images, (**b**,**d**,**f**,**h**): cipher images. (Top to down: Clock (256 × 256), Baboon (512 × 512), Peppers (512 × 512) and Male (1024 × 1024)).

In the plain image, pixels exhibit strong correlations, making them vulnerable to attacks. An ideal encryption scheme should significantly reduce these correlations, rendering the image closer to random noise. This implies that the correlation coefficient values of the encrypted image in all directions should be as close to 0 as possible. The closer the correlation coefficient is to 0, the better the encryption performance. The correlation coefficient can be calculated by Equation (20) [36]:

(20) $$\left\{\begin{array}{l} r_{xy}=\frac{E(x-E(x))(y-E(y)))}{\sqrt{D(x)}\sqrt{D(y)}}\\ E(x)=\frac{1}{Z}\sum_{i=1}^{Z}x_{i}\\ D(x)=\frac{1}{Z}\sum_{i=1}^{Z}{\left(x_{i}-E(x)\right)}^{2} \end{array}\right.,$$

where *r_xy_* is the correlation coefficient, *Z* represents the number of randomly selected pixels from the image, *E*(*x*) denotes the mean value, and *D*(*x*) represents the variance. Pixel correlation is observed between the plain images and their encrypted images in various directions. Figure 16 demonstrates the correlations of the plain images and their encrypted images in the horizontal, vertical, and diagonal directions. H, V and D on the x-axis are the horizontal, vertical, and diagonal directions, respectively. In the plain images, the grayscale values of adjacent pixels in each direction are very close, exhibiting strong correlations. However, in the cipher images, the grayscale values of adjacent pixels in each direction are distributed in a dispersed manner, demonstrating that the proposed encryption scheme effectively eliminates correlations in all directions of the image.

Table 3 shows the correlation coefficients of the plain image and the cipher image, which are analyzed in comparison with other encryption schemes. It should be noted that all algorithms involved in the comparison use the same Peppers image that was used by the proposed algorithm. As can be observed from the table, the correlation coefficients of the cipher images are significantly smaller than those of the plain images and are all close to 0. This indicates that the proposed encryption method offers significant advantages in resisting statistical attacks.

### 6.5. Information Entropy Analysis

Information entropy is used to measure the randomness of the distribution of pixel values in the image, which reflects the degree of randomness of the ciphertext image. The theoretical maximum value is log_2_(256) = 8. The closer the information entropy is to 8, the better the encryption performance, indicating that the image is closer to a random distribution. It is defined by Equation (21) [42]:

(21) $$H(X)=-\sum_{i=1}^{2^{L}-1}p(X_{i})\log_{2}p(X_{i}),$$

where *p*(*X_i_*) is the frequency of pixel value *X_i_*. Table 4 presents the entropy values of the plain images and cipher images. It can be observed that the information entropy of the cipher images is significantly higher than that of the plain images and is closer to the ideal value of 8. Furthermore, Table 4 provides a comparison of the information entropy values between the proposed algorithm and other existing algorithms. All algorithms in the comparative study were tested using the same Peppers image as the proposed algorithm. The results demonstrate that the proposed algorithm achieves slightly higher information entropy values than the existing algorithms, further validating its superiority in enhancing the security of the encrypted images.

### 6.6. Differential Attack Analysis

Differential attack analyzes the changes in the corresponding ciphertext information caused by minor modifications in the original image, revealing potential correlations between the plaintext and ciphertext. When evaluating the ability of an image encryption system to resist differential attacks, the Number of Pixel Change Rate (NPCR) and the Unified Average Changing Intensity (UACI) are two crucial metrics. Specifically, NPCR represents the percentage of pixels that have changed between the encrypted and original images, while UACI reflects the average intensity of changes in pixel values. NPCR and UACI can be calculated as follows [43]:

(22) $$NPCR(C_{1},C_{2})=\frac{\sum_{i,j}D(i,j)}{M\times N}\times 100\%,$$

(23) $$UACI(C_{1},C_{2})=\frac{1}{M\times N}\left[\sum_{i,j}\frac{\left|C_{1}(i,j)-C_{2}(i,j)\right|}{255}\right]\times 100\%,$$

(24) $$D(i,j)=\left\{\begin{array}{l} 1,C_{1}(i,j)\neq C_{2}(i,j)\\ 0,C_{1}(i,j)=C_{2}(i,j) \end{array}\right.,$$

where *C*_1_ and *C*_2_ represent the two ciphertext images, whose corresponding plain images differ by only one pixel.

In theory, since the key is generated based on the hash value of the plain image, a change in a single pixel value of the plain image will lead to a significant change in its hash value. The initial values and control parameters of the 2D-SCMH chaotic system are precisely determined by this hash value. Given that the 2D-SCMH exhibits high sensitivity to the initial values and control parameters, even a slight change in the hash value can lead to a substantial change in the chaotic sequence, thereby affecting the entire encryption process. Therefore, from a theoretical perspective, this characteristic makes differential attacks extremely difficult.

To verify that the proposed algorithm is resistant to differential attacks, we conducted experimental tests. For an image with a gray level of 256, the ideal values of NPCR and UACI are 99.6094% and 33.4635%, respectively. Table 5 shows the results of NPCR values and UACI values for the proposed algorithm and Table 6 shows the comparison of NPCR and UACI for different algorithms. As can be observed from the table, the NPCR and UACI of the proposed algorithm are very close to the theoretical ideal values and outperform those of the other algorithms. This demonstrates that the proposed algorithm can effectively resist differential attacks and exhibit strong security performance.

### 6.7. Cropping Attack

**Figure 17:**
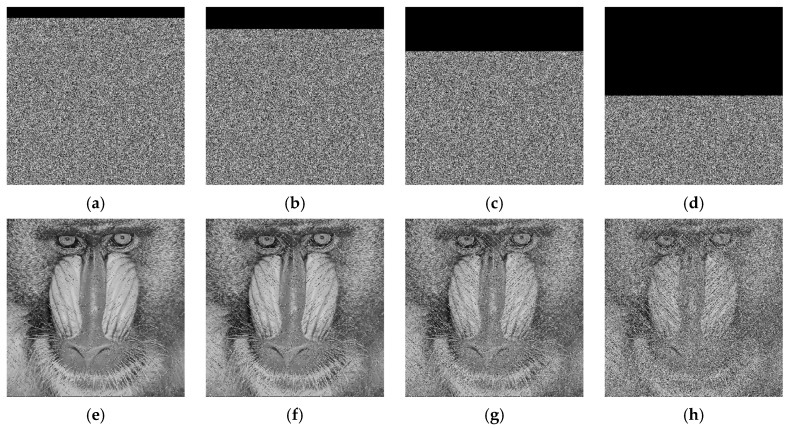
Cropping attack analyses of Baboon: (**a**) crop 1/16; (**b**) crop 1/8; (**c**) crop 1/4; (**d**) crop 1/2; (**e**) decrypted image of (**a**); (**f**) decrypted image of (**b**); (**g**) decrypted image of (**c**); (**h**) decrypted image of (**d**).

In practical transmission and storage processes, encrypted images may experience partial data loss due to various reasons. To evaluate the resistance of the proposed algorithm to cropping attacks, we performed cropping operations of different sizes on the encrypted images and then decrypted the cropped encrypted images. Figure 17 presents the experimental results of the cropping attack on the Baboon image. As can be observed from the results, although the decrypted image loses some content, the main information of the image remains recognizable.

The Peak Signal-to-Noise Ratio (PSNR) is a crucial metric for evaluating image quality. A higher PSNR value indicates less distortion and greater clarity in the image. PSNR can be calculated by Equation (25) [47]:

(25) $$\left\{\begin{array}{l} MSE=\frac{1}{M\times N}\sum_{i=1}^{M}\sum_{j=1}^{N}{\left(P_{1}(i,j)-C(i,j)\right)}^{2}\\ PSNR=10\log_{10}(\frac{255\times 255)}{MSE}) \end{array}\right.,$$

where *P* is the plain image and *C* is the cipher image. Table 7 lists the PSNR values of the decrypted images under different cropping areas. Even under large-scale cropping conditions, the PSNR remains within a certain range, demonstrating that the proposed algorithm exhibits strong robustness.

### 6.8. Noise Attack

**Figure 18:**
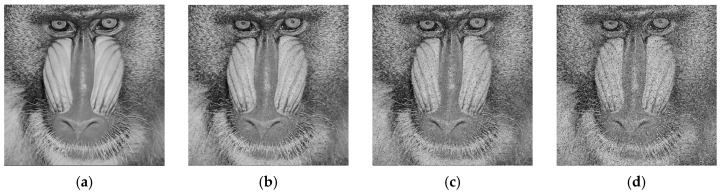
Noise attack results: (**a**) decrypted Baboon without noise; (**b**) decrypted Baboon with 0.05 noise density; (**c**) decrypted Baboon with 0.1 noise density; (**d**) decrypted Baboon with 0.2 noise density.

During the transmission or storage of images, they may be subjected to various types of noise interference. To evaluate the resistance of the proposed algorithm to noise attacks, we added salt-and-pepper noise of different intensities to the cipher image of the Baboon and conducted corresponding decryption experiments. Figure 18 presents the results for the decrypted images under different noise intensities. Experimental results show that as the noise intensity increases, the visual quality of the decrypted images gradually deteriorates. However, even under high noise intensity, the main information of the image remains recognizable. Table 8 lists the PSNR of the decrypted images under different noise intensities. As the noise intensity increases, the PSNR values remain at a relatively high level, indicating that the proposed algorithm exhibits strong resistance to noise attacks.

### 6.9. Encryption Speed Analysis

An excellent encryption algorithm should not only possess strong security but also achieve encryption in as short a time as possible. For a grayscale image of size 512 × 512, Table 9 lists the encryption times of different algorithms. Although the proposed algorithm processes each pixel individually, the entire image encryption takes only 0.4317 s due to the adoption of parallel processing technology. Compared with other algorithms, the proposed algorithm demonstrates excellent encryption efficiency.

### 6.10. Computational Complexity Analysis

Computational complexity can measure the computational resources required by an algorithm. The proposed encryption algorithm consists of three main components: chaotic sequence generation, dynamic rhombus transformation, and dynamic diffusion based on the digital tube model. Assuming the size of the encrypted image is *m* × *n*, the complexity of the chaotic sequence generation process is *O*(4 × *m* × *n*). For the dynamic rhombus transformation, the complexity is O(*m* × *n* + *L*log*L*), and since *L*log*L* ≪ *m* × *n*, the complexity of this stage remains *O*(*m* × *n*). For the dynamic diffusion algorithm based on the digital tube model, the complexity is *O*(*m* × *n*). Therefore, the overall computational complexity of the proposed encryption algorithm is *O*(6 × *m* × *n*).

## 7. Conclusions

In this paper, we proposed an image encryption algorithm based on 2D-SCMH, dynamic rhombus transformation, and a digital tube model. The dynamic rhombus transformation is used to effectively disrupt pixel positions. Meanwhile, the digital tube model is used for the diffusion operation. Experimental results and algorithm analyses demonstrate that the proposed algorithm can effectively resist various types of attacks, such as brute-force attacks, differential attacks, cropping attacks, and noise attacks. The cipher image exhibits a uniformly distributed histogram, with information entropy very close to 8. Its correlation coefficients in the horizontal, vertical and diagonal directions are 0.008, 0.0001 and 0.005, respectively. In addition, NPCR and UACI reached 99.6057% and 33.4647%, respectively, which are very close to the ideal values. Therefore, the proposed encryption algorithm can effectively protect image security in storage and transmission.

## Figures and Tables

**Table 1:** NIST test results for 2D-SCMH.

Statistical Tests	*p*_1_	*p*_2_	Result
Frequency	0.8150	0.1862	Passed
Block Frequency	0.3655	0.2725	Passed
Runs	0.8807	0.9763	Passed
Longest Run	0.3434	0.1812	Passed
Rank	0.0371	0.0554	Passed
FFT	0.5617	0.0367	Passed
Non-overlapping Template	0.5193	0.8055	Passed
Overlapping Template	0.7245	0.9468	Passed
Universal	0.9620	0.1243	Passed
Linear Complexity	0.8492	0.7126	Passed
Serial test *p*-value 1	0.3353	0.0466	Passed
Serial test *p*-value 2	0.1103	0.0459	Passed
Approximate Entropy	0.6217	0.0654	Passed
Cumulative Sums-forward	0.9996	0.4444	Passed
Cumulative Sums-reverse	0.7784	0.9117	Passed
Random Excursions Test (X = 1)	0.4360	0.9500	Passed
Random Excursions Variant Test (X = 1)	0.7255	0.1028	Passed

**Table 2:** The results of the $\chi^{2}$ test.

Image	$\mathrm{Cipher}\;\mathrm{Image}:\;\chi^{2}$	Result
Clock (256 × 256)	273.7935	Passed
Baboon (512 × 512)	273.3711	Passed
Peppers (512 × 512)	245.2773	Passed
Male (1024 × 1024)	278.0063	Passed

**Table 3:** Correlation coefficients of different images.

Plain Image	Test Image	Horizontal	Vertical	Diagonal
Clock (256 × 256)	Plain image	0.9383	0.9588	0.9736
	Cipher image	−0.0022	0.0011	−0.0022
Baboon (512 × 512)	Plain image	0.8667	0.7498	0.7158
	Cipher image	−0.0008	−0.0017	0.0014
Peppers (512 × 512)	Plain image	0.9767	0.9795	0.9625
	Cipher image	−0.0008	−0.0001	−0.0005
Male (1024 × 1024)	Plain image	0.9637	0.9776	0.9805
	Cipher image	0.0011	−0.0019	0.0017
Peppers [37]	Cipher image	0.0055	0.0026	0.0019
Peppers [38]	Cipher image	−0.0033	0.0019	−0.0088
Peppers [39]	Cipher image	0.0083	−0.0077	−0.0046
Peppers [40]	Cipher image	0.0026	−0.0037	0.0017
Peppers [41]	Cipher image	−0.0038	0.0014	−0.0036

**Table 4:** Entropy of different images.

Image	Plain Images	Cipher Images
Clock (256 × 256)	6.7057	7.9974
Baboon (512 × 512)	7.3583	7.9992
Peppers (512 × 512)	7.5821	7.9993
Male (1024 × 1024)	7.5237	7.9998
Peppers [37]	7.5821	7.9973
Peppers [38]	7.5821	7.9969
Peppers [39]	7.5821	7.9976
Peppers [40]	7.5821	7.9948
Peppers [41]	7.5821	7.9974

**Table 5:** The results of NPCR and UACI.

Image	NPCR (%)	UACI (%)
Clock (256 × 256)	99.6048	33.4681
Baboon (512 × 512)	99.6086	33.4644
Peppers (512 × 512)	99.6014	33.4621
Male (1024 × 1024)	99.6081	33.4640
Average	99.6057	33.4647

**Table 6:** Comparison of the NPCR and UACI for different algorithms.

Metric	Proposed Algorithm	Ref. [1]	Ref. [39]	Ref. [44]	Ref. [45]	Ref. [46]
NPCR (%)	99.6057	99.5893	99.5934	99.6182	99.6190	99.5899
UACI (%)	33.4647	33.3730	33.3054	33.3397	33.4931	33.6083

**Table 7:** PSNR of cropping attack.

Cropping Area	Clock (256 × 256)	Baboon (512 × 512)	Peppers (512 × 512)	Male (1024 × 1024)
1/16	19.4387	21.6272	20.0923	20.1356
1/8	16.3903	18.5846	17.9486	17.0742
1/4	13.3379	15.5898	14.9351	14.0755
1/2	10.3569	12.5724	11.9204	11.0361

**Table 8:** PSNR of noise attack.

Noise Intensity	Clock (256 × 256)	Baboon (512 × 512)	Peppers (512 × 512)	Male (1024 × 1024)
0.05	20.2177	22.5324	21.9483	21.0056
0.1	17.3948	19.5341	18.8583	18.0037
0.2	14.2832	16.5614	15.8815	14.9865

**Table 9:** Comparison of encryption time among different algorithms.

Algorithm	Proposed Algorithm	Ref. [16]	Ref. [48]	Ref. [49]	Ref. [50]	Ref. [51]
Time (s)	0.4317	0.4984	1.68	1.6005	1.2065	1.5181

## Data Availability

Some or all of the data generated or used in this study are available from the corresponding author upon reasonable request.
